# Long‐term Endoscopic Evolution of a Pyloric Gland Adenoma Arising in Nonatrophic Gastric Mucosa and Initially Resembling a Fundic Gland Polyp

**DOI:** 10.1002/deo2.70464

**Published:** 2026-11-04

**Authors:** Ryohei Kobayashi, Hiroyuki Nakanishi, Kenta Takaura, Shohei Tanaka, Chiaki Maeyashiki, Nobuharu Tamaki, Yutaka Yasui, Urara Sakurai, Kaoru Tsuchiya, Masayuki Kurosaki

**Affiliations:** ^1^ Department of Gastroenterology and Hepatology Musashino Red Cross Hospital Tokyo Japan; ^2^ Department of Diagnostic Pathology Musashino Red Cross Hospital Tokyo Japan

**Keywords:** endoscopic submucosal dissection, fundic gland polyp, nonatrophic gastric mucosa, pyloric gland adenoma, serial endoscopic observation

## Abstract

Pyloric gland adenoma (PGA) is a rare gastric epithelial neoplasm often associated with autoimmune or metaplastic atrophic gastritis. An 80‐year‐old man underwent serial upper gastrointestinal endoscopy. A small gastric fundus lesion initially resembling a fundic gland polyp (FGP) was followed without biopsy. Over 11 years, it enlarged to 22 mm and became increasingly whitish, against a background described endoscopically as nonatrophic. Biopsy suggested PGA, and the lesion was resected en bloc by endoscopic submucosal dissection. Closely packed pyloric‐type glands showed elongation, tortuosity, branching, and cystic dilatation, with diffuse MUC6 expression and MUC5AC expression restricted to the surface epithelium. The lesion was diagnosed as PGA with low‐grade dysplasia. The adjacent oxyntic mucosa showed no atrophy, lymphoplasmacytic infiltration, or nodular enterochromaffin‐like cell hyperplasia. Without an initial biopsy, whether the lesion was already PGA or developed within a pre‐existing FGP remains unknown. An enlarging or increasingly whitish FGP‐like lesion warrants histologic assessment, even in nonatrophic gastric mucosa.

## 1 Introduction

Pyloric gland adenoma (PGA) shows differentiation toward pyloric glands and mucous neck cells. Most gastric PGAs arise in the corpus or fundus and are associated with autoimmune gastritis (AIG) or metaplastic atrophic gastritis, although they also occur in normal mucosa [1, 2, 3, 4]. Recognition is important because PGA may contain high‐grade dysplasia or adenocarcinoma [1, 4].

The endoscopic appearance of PGA varies and can resemble a non‐neoplastic gastric polyp [5]. Longitudinal observation of duodenal PGA arising from heterotopic gastric mucosa has been reported [6], but the course of gastric lesions before histologic diagnosis remains poorly documented. We describe a fundic gland polyp (FGP)‐like lesion in nonatrophic gastric mucosa that enlarged and became whitish over 11 years before being diagnosed as PGA.

## 2 Case Report

An 80‐year‐old man underwent regular screening upper gastrointestinal endoscopy. Eleven years before resection, a small, smooth, sessile elevation was identified in the gastric fundus (Figure 1a). It resembled multiple FGP‐like lesions in the fundus and corpus and was followed without biopsy. The background mucosa was endoscopically assessed as nonatrophic. There was no history of long‐term proton pump inhibitor use or *Helicobacter pylori* eradication. The urea breath test was negative, but serum anti‐*H. pylori* IgG was mildly elevated at 13 U/mL; previous infection could not be definitively excluded. There was no family history suggestive of hereditary polyposis, and colonoscopy showed no colorectal polyposis.

**FIGURE 1:**
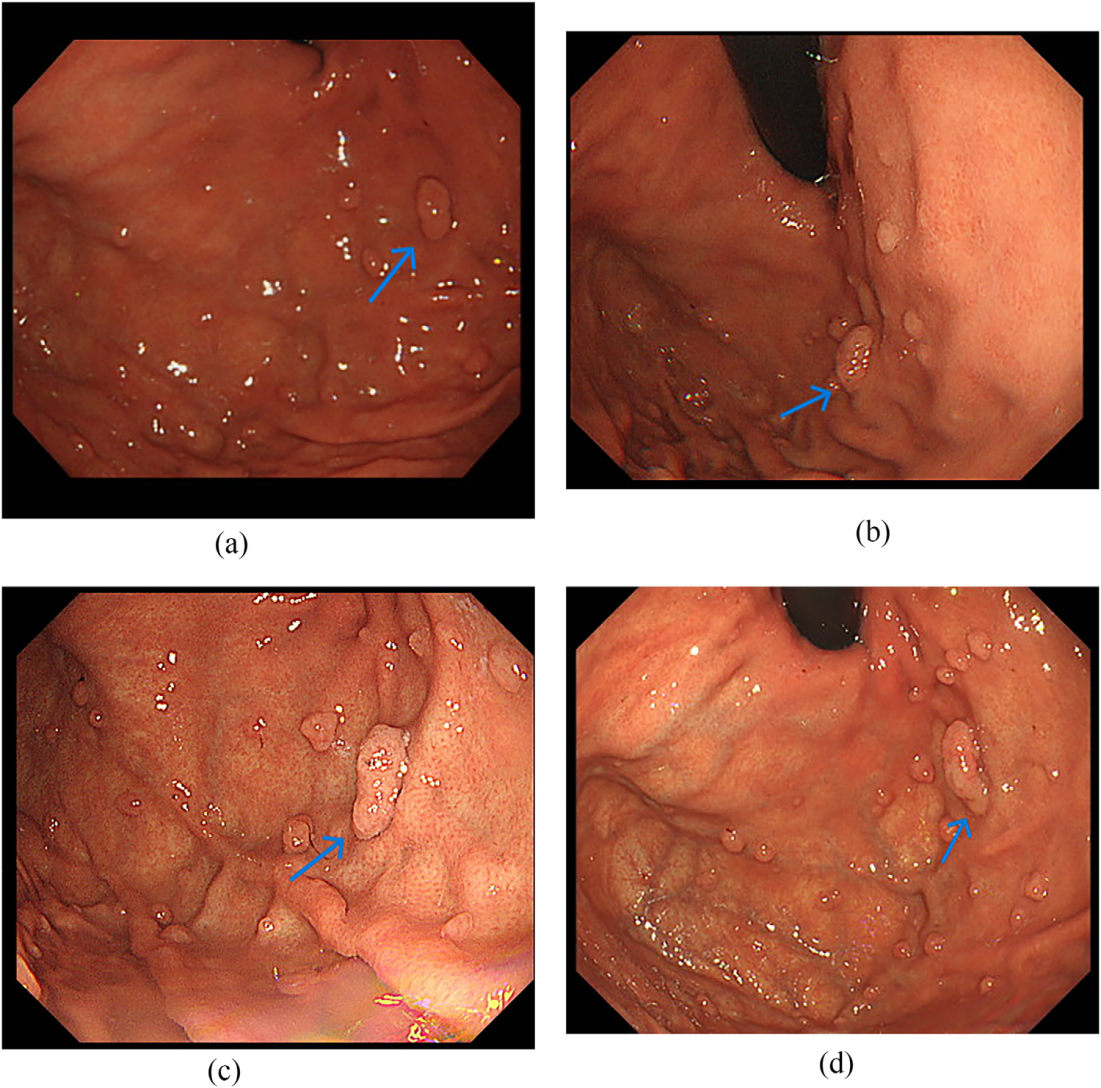
Long‐term endoscopic changes in the gastric lesion. White‐light images show gradual enlargement of the fundic gland polyp‐like lesion: (a) 5 mm 11 years before resection, (b) 8 mm 7 years before resection, (c) 12 mm 3 years before resection, and (d) 15 mm 1 year before resection. Progressive whitish discoloration is also evident.

Anti‐parietal cell antibodies were positive at a titer of 1:10, and anti‐intrinsic factor antibodies were negative. Hemoglobin was 13.4 g/dL, serum gastrin was 78.1 pg/mL (institutional upper limit, 46.9 pg/mL), and serum vitamin B12 was 317 pg/mL. Serum iron, ferritin, pepsinogen I, and pepsinogen II were not measured; the pepsinogen I/II ratio was therefore unavailable. The patient had no history of autoimmune thyroid disease, including Hashimoto's thyroiditis, or other autoimmune disorders.

Endoscopy 7, 3, and 1 years before resection showed gradual enlargement and progressive whitish discoloration (Figure 1b–d). At the time of resection, the lesion measured 22 × 17 mm and had a whitish, irregular surface with a 0‐I+IIa‐like morphology (Figure 2a–d).

**FIGURE 2:**
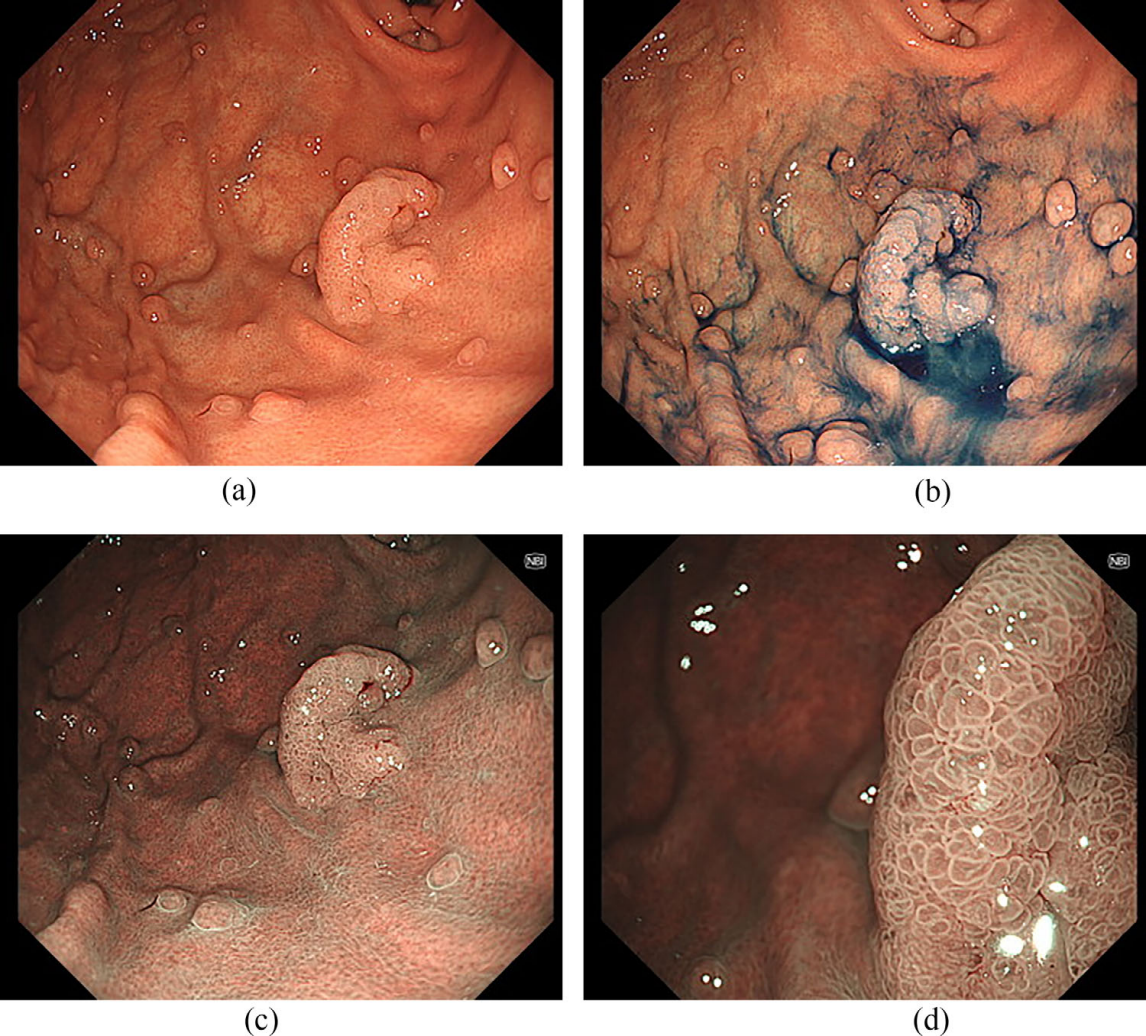
Endoscopic findings at the time of resection. (a) White‐light imaging shows a 22‐mm whitish elevated lesion in the gastric fundus. (b) Indigo carmine chromoendoscopy delineates the lesion margin and surface morphology. (c) Narrow‐band imaging (NBI) enhances the contrast between the lesion and the surrounding mucosa. (d) Close‐up NBI reveals the surface pattern and microvascular architecture.

Biopsy showed foveolar hyperplasia and atypical superficial glands, leading to a diagnosis of gastric adenoma. The glands were positive for MUC6, focally positive for MUC5AC, negative for MUC2, and weakly positive for pepsinogen I, with a few admixed H+/K+‐ATPase‐positive parietal cells. PGA was suspected, and complete endoscopic resection was selected for diagnosis and treatment.

The lesion was resected *en bloc* by endoscopic submucosal dissection (ESD) without adverse events. Surveillance endoscopy 1 year after resection showed no local recurrence (Figure 3). The specimen measured 35 × 28 mm and contained a well‐demarcated, 22 × 17‐mm lesion. Closely packed neoplastic glands were elongated, tortuous, branched, and cystically dilated, consisting mainly of columnar mucinous cells with focal deeper parietal and chief cell differentiation (Figure 4a). MUC6 was diffusely positive, MUC5AC expression was restricted to the surface epithelium (Figure 4b,c), MUC2 was negative, and pepsinogen I was weakly positive. The predominantly tubular architecture, preserved nuclear polarity, low Ki‐67 labeling index, and wild‐type p53 pattern supported PGA with low‐grade dysplasia according to the fifth edition of the World Health Organization (WHO) Classification of Tumors. Horizontal and vertical margins were negative, and no ulceration was present.

**FIGURE 3:**
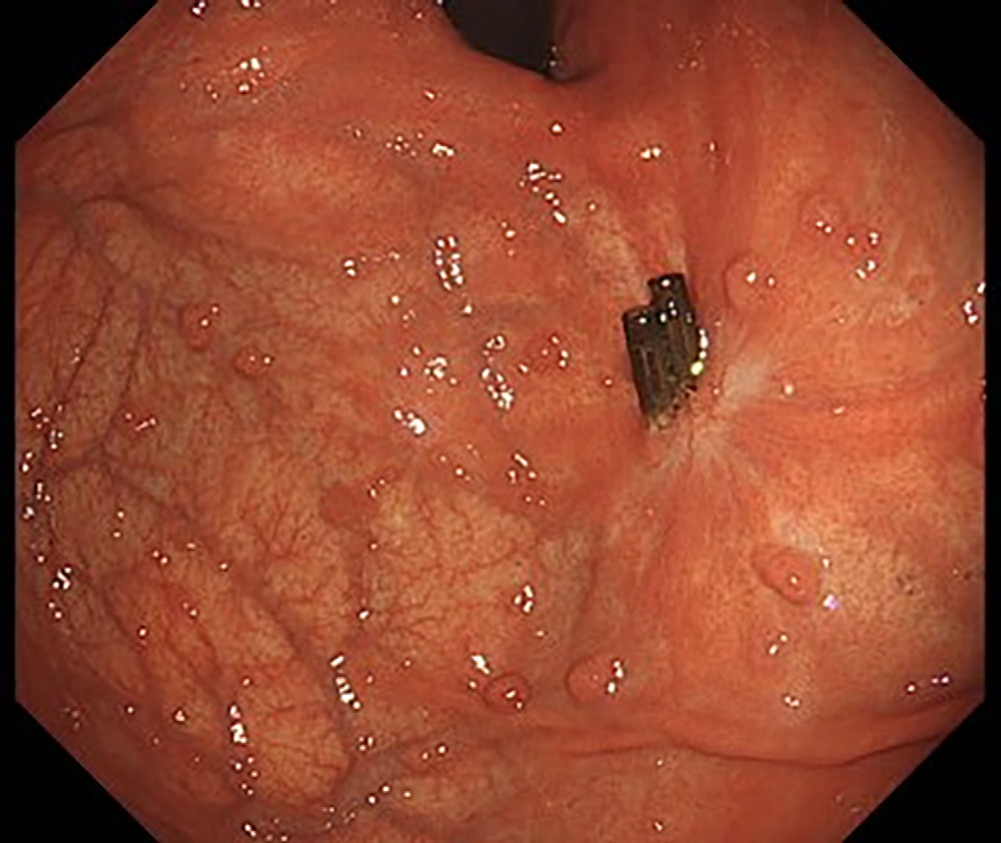
Surveillance endoscopy 1 year after resection shows a scar in the gastric fundus without a residual or recurrent lesion.

**FIGURE 4:**
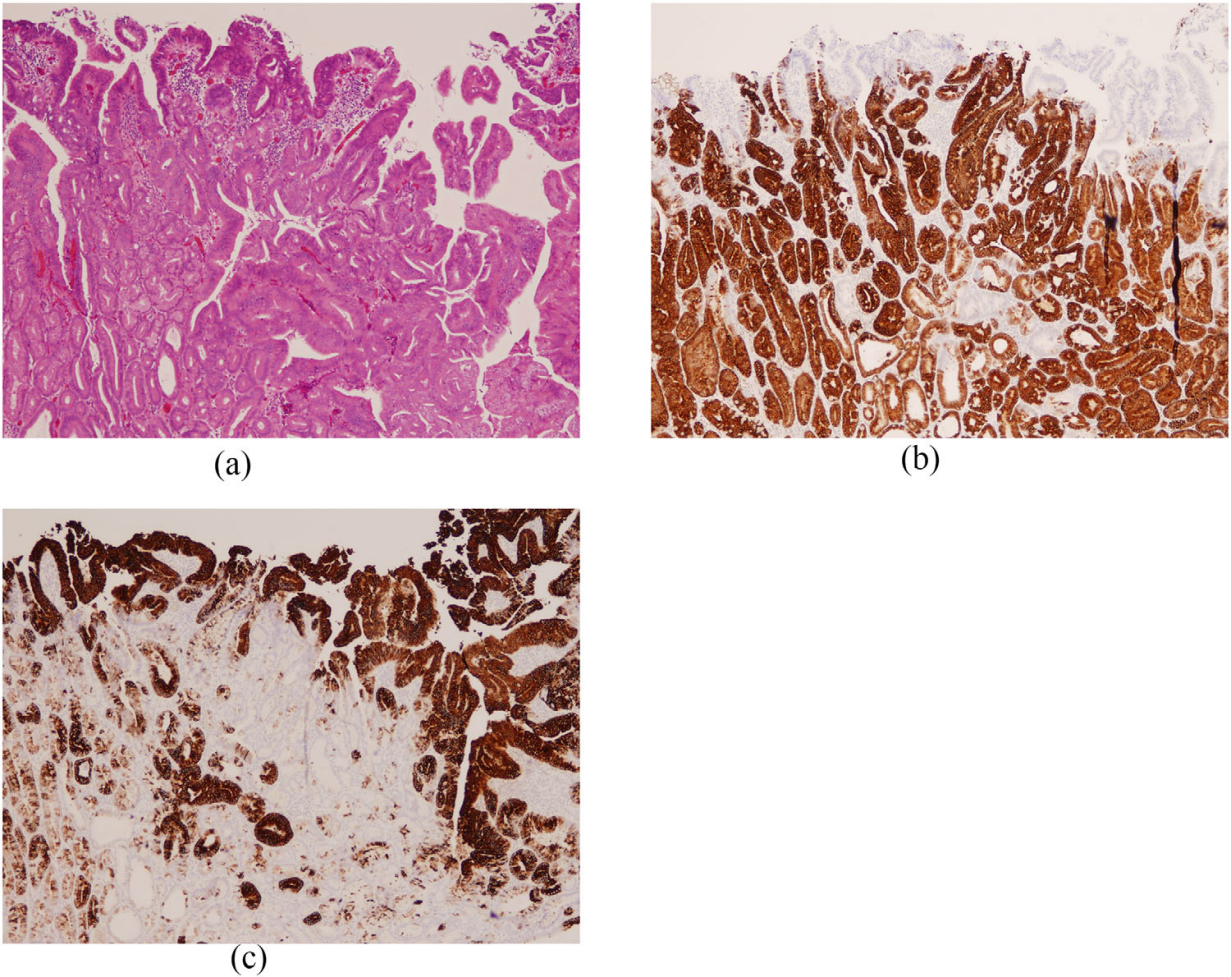
Histopathologic and immunohistochemical findings. (a) Hematoxylin and eosin staining shows closely packed glands with elongation, tortuosity, branching, and cystic dilatation (×40). (b) MUC6 is diffusely expressed in the cytoplasm of the neoplastic glands (×40). (c) MUC5AC expression is restricted to the surface epithelium (×40).

Histologic assessment of the background mucosa was limited to the non‐neoplastic oxyntic mucosa adjacent to the lesion in the ESD specimen. No separate biopsies of the background antral or corpus mucosa were obtained. The sampled mucosa showed no glandular atrophy or lymphoplasmacytic infiltration. Chromogranin A immunostaining showed no nodular enterochromaffin‐like cell hyperplasia.

## 3 Discussion

This case documents 11 years of endoscopic observation of an FGP‐like lesion subsequently diagnosed as PGA. The observed changes were gradual enlargement and progressive whitish discoloration. Because no initial biopsy was obtained, whether the lesion was already PGA or developed within a pre‐existing FGP remains unknown. Neither possibility can be established or excluded by the serial images.

PGA can arise without gastric atrophy. In a multicenter series of 67 gastric PGAs from 57 patients, 35.8% arose in normal mucosa [4]. In this case, the negative urea breath test and nonatrophic background did not establish *H. pylori*‐naive status because serum anti‐*H. pylori* IgG was positive.

The anti‐parietal cell antibody titer of 1:10 was interpreted as positive. AIG requires assessment of autoantibodies together with endoscopic and histologic findings [7]. Serum gastrin was mildly elevated, but the adjacent oxyntic mucosa showed no histologic findings supporting AIG. We considered the apparently atrophic appearance after resection to reflect scarring associated with healing of the post‐ESD ulcer. This appearance alone does not establish progressive atrophy of the background mucosa. Because no separate antral or corpus biopsies were obtained, early or patchy AIG elsewhere in the stomach cannot be completely excluded.

Progressive enlargement and whitish discoloration helped distinguish this lesion from the surrounding FGP‐like elevations and prompted biopsy. These changes may be useful warning signs, although their histologic mechanism cannot be determined from this single case.

Nonaka et al. described a polyp containing conventional FGP, cystically altered FGP, and PGA components with shared and stepwise APC, GNAS, and KRAS alterations, supporting a histogenetic relationship [8]. Our case lacked initial histology and molecular analysis. Progression from an FGP to PGA therefore remains possible but was not demonstrated.

Closely packed pyloric‐type glands, diffuse MUC6 expression, and superficial MUC5AC expression supported the diagnosis of PGA [2, 3]. Chief and parietal cells formed only a minor deeper component, and pepsinogen I was weakly expressed. The combined architecture and differentiation pattern favored PGA over an oxyntic gland neoplasm; weak pepsinogen I staining alone does not establish this distinction. Low‐grade dysplasia was supported primarily by the preserved nuclear polarity and tubular architecture, with low Ki‐67 and wild‐type p53 providing additional support.

Ueyama et al. have proposed an endoscopically anchored framework for gastric epithelial neoplasms (GENs) arising in *H. pylori*‐uninfected stomachs, in which the endoscopic category of whitish flat elevated‐type GEN encompasses lesions whose histologic counterparts include foveolar‐type adenoma and PGA; the authors note that the pathologic definitions within this category remain unestablished [9]. Endoscopically, the present lesion shares the principal features of this category: a whitish, well‐demarcated elevated lesion arising in nonatrophic fundic mucosa. Histologically, closely packed, elongated, tortuous, branched, and cystically dilated pyloric‐type glands predominated, with diffuse MUC6 expression and MUC5AC restricted to the surface epithelium—features of a lesion arising beneath the foveolar surface rather than of a foveolar‐phenotype surface dysplasia. In the sixth edition of the WHO classification, the sections previously dedicated to gastric dysplasia and to intestinal‐ and gastric‐type adenomas have been combined under gastric dysplasia, whereas lesions arising beneath the gastric foveolar surface, including oxyntic gland neoplasms and PGA, are addressed separately [10]. We therefore retain PGA as the histopathologic diagnosis, while acknowledging that this lesion falls within the endoscopic category of whitish flat elevated‐type GEN and that the relationship between the two frameworks awaits consensus.

Complete histologic assessment is appropriate because PGA may contain high‐grade dysplasia or carcinoma [1, 4]. Reported frequencies in resected series should not be interpreted as prospective malignant transformation rates. En bloc ESD allowed definitive assessment of this enlarging 22‐mm lesion. An FGP‐like lesion that enlarges or becomes increasingly whitish warrants biopsy and consideration of complete resection, even in nonatrophic mucosa.

## Funding

The authors have nothing to report.

## Ethics Statement

Institutional review board approval was not required for this single case report.

## Consent

Written informed consent was obtained from the patient for publication of this report and the accompanying images.

## Conflicts of Interest

The authors declare no conflicts of interest.

## Data Availability

All data relevant to this manuscript are included in the article. Additional information is available from the corresponding author upon reasonable request.
